# Enhancing sampling design in mist-net bat surveys by accounting for sample size optimization

**DOI:** 10.1371/journal.pone.0174067

**Published:** 2017-03-23

**Authors:** Leonardo Carreira Trevelin, Roberto Leonan Morim Novaes, Paul François Colas-Rosas, Thayse Cristhina Melo Benathar, Carlos A. Peres

**Affiliations:** 1 Programa de Pós-graduação em Zoologia, Museu Paraense Emílio Goeldi/ Universidade Federal do Pará, Belém, PA, Brazil; 2 Fiocruz Mata Atlântica, Fundação Oswaldo Cruz, Rio de Janeiro, RJ, Brazil; 3 Biophilium Consultoria Ambiental Ltda, Atibaia, SP, Brazil; 4 Laboratório de Citogenética, CEABIO, ICB, Universidade Federal do Pará, Belém, Pará, Brazil; 5 Centre for Ecology, Evolution and Conservation, School of Environmental Sciences, University of East Anglia, Norwich, United Kingdom; Università degli Studi di Napoli Federico II, ITALY

## Abstract

The advantages of mist-netting, the main technique used in Neotropical bat community studies to date, include logistical implementation, standardization and sampling representativeness. Nonetheless, study designs still have to deal with issues of detectability related to how different species behave and use the environment. Yet there is considerable sampling heterogeneity across available studies in the literature. Here, we approach the problem of sample size optimization. We evaluated the common sense hypothesis that the first six hours comprise the period of peak night activity for several species, thereby resulting in a representative sample for the whole night. To this end, we combined re-sampling techniques, species accumulation curves, threshold analysis, and community concordance of species compositional data, and applied them to datasets of three different Neotropical biomes (*Amazonia*, *Atlantic Forest* and *Cerrado*). We show that the strategy of restricting sampling to only six hours of the night frequently results in incomplete sampling representation of the entire bat community investigated. From a quantitative standpoint, results corroborated the existence of a major Sample Area effect in all datasets, although for the *Amazonia* dataset the six-hour strategy was significantly less species-rich after extrapolation, and for the *Cerrado* dataset it was more efficient. From the qualitative standpoint, however, results demonstrated that, for all three datasets, the identity of species that are effectively sampled will be inherently impacted by choices of sub-sampling schedule. We also propose an alternative six-hour sampling strategy (at the beginning and the end of a sample night) which performed better when resampling *Amazonian* and *Atlantic Forest* datasets on bat assemblages. Given the observed magnitude of our results, we propose that sample representativeness has to be carefully weighed against study objectives, and recommend that the trade-off between logistical constraints and additional sampling performance should be carefully evaluated.

## Introduction

Mist-netting remains as one of the most effective methods of sampling flying vertebrates. Since its introduction as a formal sampling protocol, nets revolutionized wildlife studies, and most notably bat research [1, 2]. Advantages of mist-nets are their ease in deployment, relatively low cost, and portability [3]. Field studies of bat communities employ mist-nets as the main sampling technique, particularly across the Neotropical realm [4] as bats are difficult to be visually detected due to often strictly nocturnal habits. Moreover, indirect sampling and monitoring based on acoustic techniques, such as ultrasonic digital recording, still depend on ongoing technological and empirical advances before they can become reliable and widespread [5]. Yet, direct sampling of microchiropteran assemblages over long field periods pose intrinsic difficulties for even the most enthusiastic nocturnal investigators, not least because the evolutionary legacy of the human sensory apparatus harks back to the earliest diurnal primates. It is therefore understandable that fieldworkers should attempt to minimize time invested in nocturnal surveys.

In the Neotropics, bats often comprise the most abundant, species-rich and ecologically diverse mammalian fauna at the local scale (alpha diversity), posing an enormous challenge in properly investigating this diversity [6]. Ideally, several complementary techniques should be used to fully explore a Neotropical bat assemblage [1,2,3]. However, a quantitative sampling protocol based on the sole use of mist-nets can be justified as ensuring logistically feasible data quality control on the standardization and representativeness of any focal assemblages [2,5]. While more representative of any studied Neotropical assemblage compared to alternative methodologies, mist-net sampling study designs still have to deal with issues of detectability related to how different species behave and use the environment, in attempting to capture the entirety or a standardized portion of this heterogeneity [5, 7]. Sources of heterogeneity include vertical and horizontal foraging stratification of different species [8–10], and temporal activity stratification during a single night [11]. Sampling designs that deal with this heterogeneity may however substantially increase research costs, leading researchers to face the crossroads between study representativeness and feasibility [12].

In an interesting study conducted by Marques and collaborators [13], the authors proposed optimization guidelines when designing sampling strategies with mist-nets. By demonstrating the effects of net avoidance by bats and birds due to spatial learning of net locations, they evaluated the negative effects of this form of sampling bias on survey efficiency, particularly for bats. Their main conclusion is that moving nets each day would be important to minimize net shyness of bats only if this approach does not represent overall loss of net time during any given expedition. If so, either investing time in moving nets on a daily basis or incurring sampling losses due to net shyness would “cost” roughly the same amount of sampling efficiency. While we completely agree with their elegant demonstration of the effects of net shyness, we believe their results for different sampling strategies remain incomplete for bat field studies, and that this discussion can benefit from understanding the variation in sampling efficiency during nocturnal surveys [14].

Here, we critically evaluate the common sense notion that pervades the vast majority of mist-netting bat studies in the Neotropics: the first six hours comprise the activity peak for most species during the night, thereby presumably yielding a representative sample of the entire sampling night [10, 15–19] (even when this goes against previous evidence [14, 20]). To this end, we combined re-sampling techniques and species accumulation curves [21, 22] with an approach based both on community concordance of compositional data [12, 23] and also Threshold Indicator Taxa ANalysis (TITAN) to identify thresholds at both individual species and community level along an entire night-time gradient [24]. We attempted to isolate sampling area effects (i.e. larger samples include more individuals and, for any given abundance distribution, more species) from patterns of species and functional group composition related to species activity patterns [11, 25]. This analytical approach was employed to evaluate existing datasets from three different Neotropical forest and scrubland biomes within Brazil (*Amazonia*, *Atlantic Forest* and *Cerrado*). Finally, we draw conclusions to complement the sampling guidelines proposed by Marques and collaborators [13].

## Materials and methods

### Study areas

**Fig 1:**
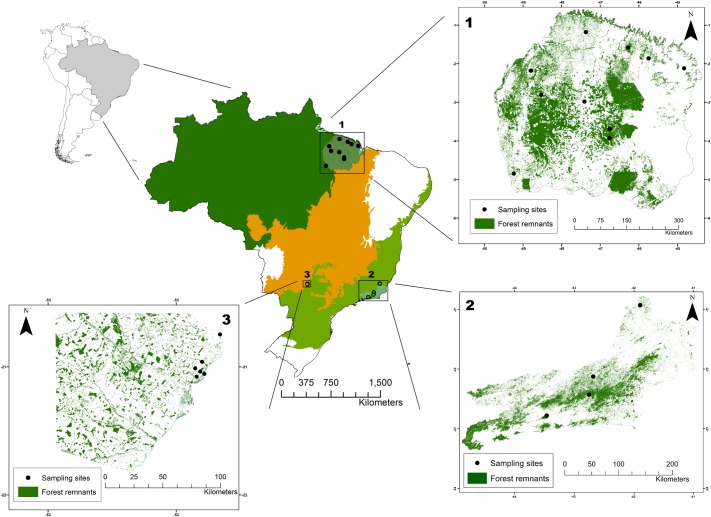
Location map. Location of the study region of all three datasets. Dark green, light green and orange indicate the Amazon forest biome, the Atlantic forest biome, and the Brazilian Cerrado biome, respectively. 1: Amazonia dataset; 2: Atlantic Forest dataset; 3: Cerrado dataset. Source data used for this map was downloaded from MapBiomas [30].

Three datasets from different Brazilian biomes were assembled to examine the questions proposed in this study (Fig 1). The *Amazonia* dataset corresponds to a previously unpublished metacommunity study by LCT conducted in an eastern Amazonian interfluvial region, which in the biogeographic literature is referred to as the Belém Endemism Area. Located between the Tocantins and Mearin rivers, this region coincides with the longest history of modern human occupation across the Amazon, and retains only ~24% of its original dense evergreen upland (terra firme) and seasonally-flooded (igapó and várzea) forests [26, 27]. Forest remnants are scattered across fragmented and variegated landscapes, and this dataset comprises 10 bat communities sampled over 66 nights, resulting in 1,742 individuals captured between 2010 and 2014.

The *Atlantic Forest* dataset comprises four bat communities sampled in fragmented landscapes of the Atlantic Forest of the state of Rio de Janeiro. Sampling was conducted between 2008 and 2012, and encompassed dense evergreen forests at the continental boundaries between the Serra do Mar and seasonal semi-deciduous forests, which represent typical low to mid elevation forests of coastal regions of southeastern Brazil. Data from these four bat communities represent 59 sampling nights and 1,877 individuals captured [28, 29].

The *Cerrado* dataset corresponds to metadata of wildlife monitoring studies conducted at locations on the east bank of the Paraná river, state of Mato Grosso do Sul. This region encompasses all vegetation physiognomies of the Central Brazilian scrublands (*cerrado* sensu lato), including tropical grasslands, savannahs and seasonally dry forests, all of which embedded within a matrix of pastures and *Eucalyptus* plantations. Data were collected across six remnants of native vegetation, amounting to 24 nights and 489 individuals captured.

### Data sampling

Bat sampling across all datasets was carried out using understory mist-nets placed along trails within forest areas, at the edge of forest remnants, and near waterbodies. Each sampling replicate consisted of approximately 12 consecutive hours of sampling, from sunset (approx. 18.00h) to sunrise (approx. 06.00h), when nets remained open and were checked every 30 min over the entire night. Each sampling night consisted of five 12 m x 2.5 m nets in the *Amazonia* dataset, and five to eight 12 m x 2.5 nets in the *Atlantic Forest* and *Cerrado* datasets. These procedures were repeated at least once during both the wet and dry seasons for all localities in all three datasets.

We recorded the time of capture of all sampled individuals, which were sexed, weighted and identified in the field, on the basis of specialized keys and publications [31, 32], and a collective amount of 54 person-years of bat fieldwork experience across all three biomes. The majority of individuals were released in the same site of capture, but voucher specimens were occasionally collected and deposited in the mammal collections of the Museu Paraense Emilio Goeldi (MPEG), Museu Nacional (MN-UFRJ) and Museu de Zoologia of the University of São Paulo (MZUSP), for the Amazonian, the Atlantic Forest and the Cerrado datasets, respectively. Fieldwork, handling and processing of all captures at all study sites were in compliance to the guidelines of the American Society of Mammalogists [33] and AVMA Guidelines for the Euthanasia of Animals [34], and were authorized by the appropriate Brazilian authority, namely the Brazilian Instituto Chico Mendes de Biodiversidade (ICMBio) (SISBIO licenses: 24463; 29722; 50337; 3893-1/28717; 1896-1/15809).

Captures obtained during each night sample (replicates) where pooled within its respective dataset, resulting in a profile of bat activity for that region. Since comparisons were performed within, rather than between, datasets, standardization in net numbers per night was ensured. Despite occasional captures of other families, datasets compiled for this study were comprised only of Phyllostomid and Vespertilionid species (98% and 02% of all captures across biomes, respectively), which are regularly captured with the implemented methodology, and thereby ensuring the desired standardization [2,3].

### Data analysis

#### (i) Study design

*Are the first six hours of a sampling night representative samples from the whole night of activity in Neotropical bat communities*? We first postulated that a positive answer to this central question of the study would imply that a sample within the first six hours of the night (18.00h – 24.00h) comprises the peak of bat activity for all species during an entire night. If so, patterns in species, rarity and functional richness and composition of the entire species assemblage should be quantified using this sampling strategy. Next, we hypothesized that two main effects could be influencing this scenario: (i) the sample area effect (SA), a simple null model that predicts that larger sample areas will contain more individuals and likely more species for any given abundance distribution [25]; and (ii) the differential species activity pattern (DSAP) effect, our biological model that predicts that species are asynchronous in their inherent activity patterns accordingly to specific natural histories, resulting in different species activity peaks throughout the nocturnal cycle [11]. Evidently, the former implies a positive confirmation to our proposed question, in which differences in ecological responses only reflects differences in sample sizes, while the latter implies a biological effect related to the foraging ecology of each species. The analytical scheme proposed to answer this question was applied to all three datasets independently.

#### (ii) Rarity and functional characterization

We accessed rarity patterns of all species recorded in each dataset, based on the local and regional abundance, and geographic range. Since these are complementary dimensions of rarity that determine species extinction risk [35, 36], they were integrated into a framework to access patterns of species´ commonness versus rarity. The local abundance of a species *i* (LA_*i*_) was determined as the median of the relative abundance of that species obtained for each sampling site (the mean number of records of that species during each replicate night divided by total number of records for that site). The regional abundance of a species *i* (RA_*i*_) was determined as the total number of records obtained for that region (dataset), divided by the total number of sampling sites of species *i*. For the geographic range of each species (GR_*i*_), we generated estimates based on our own datasets as distributional boundaries of several study species are still poorly determined [36, 37]. To this end, we estimated the total area (km^2^) of the minimum convex polygon encompassed by the outermost limits of occurrence of each species, as defined by our samples in all three datasets. The three metrics were then combined into one single rarity index (RI*i*), as in the study of Leitão and collaborators [36]. Each metric was first log-transformed and standardized between 0.0–1.0, and thus the index was calculated as

$${RI}_{i}=\frac{\left[({LA}_{i}\times \omega_{LA})+({RA}_{i}\times \omega_{RA})+({GR}_{i}\times \omega_{GR})\right]}{\left(\omega_{LA}+\omega_{RA}+\omega_{GR}\right)},$$

where *ω*_*LA*_, ω_*RA*_ and *ω*_*GR*_ are the weighting parameters that represent the degree of independence between each rarity metric and the others. These were calculated for all three metrics following the example for local abundance:

$$\omega_{LA}=\frac{1}{2}+\left[(\frac{1-|r_{LARA}|}{2})+(\frac{1-|r_{LAGR}|}{2})\right],$$

where *r*_*LARA*_ is the Pearson correlation coefficient between the local abundance and regional abundance; and *r*_*LAGR*_ is the Pearson correlation coefficient between the local abundance and the geographic range [36]. Using RI estimates, the final step was to classify each species into Common (C), Uncommon (U) or Rare (R), following the quartile method [35]. As noted by Leitão and collaborators, our RI is also a context-dependent metric, whereby the same species can be classified as rare in one dataset but common in another [36].

Functional groups in this study were defined using a qualitative approach, where species were characterized following the seminal work of Kalko, Handley Jr. and Handley [38]. In their study, 66 species recorded in a long-term study in Costa Rica were classified into guilds based on habitat type, foraging mode and diet. All species recorded in our datasets could be successfully assigned to one of the proposed guilds (same species or geographic equivalent of the same genus) and, as we restricted the analysis only to Phyllostomids and Vespertilionids, seven guilds were annotated: Background Cluttered Space—Aerial Insectivores (AI), Highly Cluttered Space—Gleaning Insectivores (GI), Highly Cluttered Space—Gleaning Carnivores (CA), Highly Cluttered Space—Gleaning Sanguivores (SA), Highly Cluttered Space—Gleaning Frugivores (FR), Highly Cluttered Space—Gleaning Nectarivores (NE), and Highly Cluttered Space—Gleaning Omnivores (OM).

#### (iii) Analytical procedures

To evaluate general activity patterns, the relative frequency of individuals recorded over time was plotted as stacked bar graphs. Three graphs were generated for each dataset, one representing each different bat species, one classifying species into their rarity categories and the other into functional categories. We then applied the Threshold Indicator Taxa Analysis (TITAN) to identify abrupt changes in both the frequency and relative abundance of each species along the nocturnal chronosequence (i.e. 12-hour comprising a full night) and assess the relative synchrony among these change points to detect community-wide thresholds [24]. For each species recorded, midpoints between all observed times of captures were considered candidate change points used to iteratively split observations into two groups, and calculate IndVal (Indicator Value) scores for each group (negative and positive associations to the left and right of the change point) [39]. The greatest IndVal score and its direction of association were kept for comparison with other candidate change points. Confidence intervals of change point locations were determined through a bootstrap, and each species was considered to respond either positively or negatively along the time gradient if observed changes were in the same direction for at least 95% of all bootstrapped runs (i.e. high purity), and at least 95% of all bootstrapped runs were significantly different from a random distribution (i.e. high reliability) [24]. Indicators of community-level thresholds corresponded to peaks in sums of all z scores along the gradient associated either with the maximum decline for all negative responders (z‒) or increase in frequency and abundance for all positive responders (z+). This analysis was performed considering the minimum split size of four (*minSplt*) and species with less than nine records were grouped by guild and rarity categories [24]. We analyzed TITAN´s results in relation to the rarity and functional attributes of each species seeking possible associations between these attributes and detection thresholds along the 12-h time gradient, an initial approach to examine DSAP effects [40].

The original species occurrence matrices (12-h sampling strategy) were then sub-divided into matrices that corresponded to those same sampling events but assuming that only the first six hours of activity had been sampled (hereafter, six-hour sampling strategy). We further outlined a novel, alternative third sampling strategy, whereby nets were opened during the first three and the last three hours of the nocturnal cycle, aiming to sample different activity peaks (hereafter, six-hour-B sampling strategy).

The SA effect was mainly addressed using abundance and species richness pattern analysis. To this end, we performed comparisons of both sample-based and individual-based rarefaction curves, calculated analytically using EstimateS 9.1.0 [21, 41]. Rarefaction curves were extrapolated to the total number of samples (or individuals) of each dataset based on the Bernoulli product model [22].

The DSAP effect was also examined through similarity analysis of community composition [12, 23]. A Principal Coordinates Analysis (PCoA) ordination was computed twice for all sampling strategies, using both the Jaccard and Bray-Curtis measures of dissimilarity in community space. The former was used after transformation of all matrices into presence/absence data, and was meant to emphasize only changes in species composition, whereas the latter integrates both species composition and abundance [12, 42]. Configuration of site scores along the ordination axes represent the patterns of species composition of each sampling strategy [42]. Congruence between species composition was examined using Procrustes rotation analysis, where an algorithm is used to minimize the sum of squared residuals between any two compared matrices. The procedure results in a *m*^*2*^ statistic which, once transformed into the *r* statistic = sq-rt of 1-*m*^*2*^), is considered a robust measure of the level of community congruence [43]. The statistical significance of all *r-*statistics generated was assessed by randomization tests with the PROTEST routine [44]. We used the first five axes of the PCoAs for all comparisons, which accounted for a substantial proportion of the overall variation in the datasets (S1 Table).

Finally, to evaluate whether the performance of each predetermined sampling strategy relied more heavily on either the biological effect or the sampling area effect, we repeated all steps of the community composition similarity analysis using null models. These null models consisted of 1,000 sub-samples that were randomly drawn from original dataset, with increasing numbers of randomly selected species. Null-model results (*r*-statistics) were then plotted together with the results of each sampling strategy compared in this study. Except where noted, all analyses were conducted within the R environment [45], using the *vegan*, *titan2* and *lattice* packages [46, 47, 48].

## Results

### Overall activity patterns

**Fig 2:**
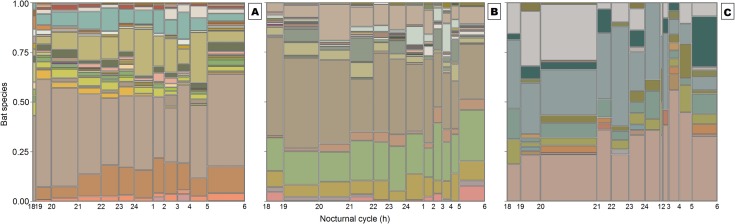
**Mosaic plot representing the cross-sectional distribution of bat species sampled chronologically using mist-nets during the entire nocturnal cycle (18:00h – 06:00h) for the datasets derived from the (A) Amazon, (B) Atlantic Forest and (C) Cerrado biomes.** Color-coded areas of the stacked tiles, or bin sizes, represent different bat species and are proportional to the number of bat captures within sequential time categories.

**Fig 3:**
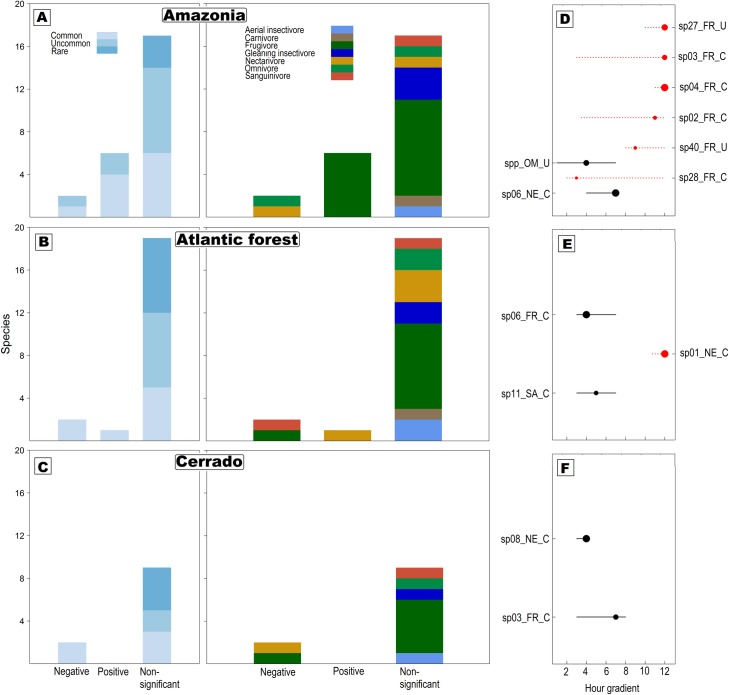
Species responses in TITAN sorted by rarity and functional attributes. (A-B) Proportions of species with negative, positive and non-significant thresholds, and classed in terms of both rarity and functional groups. (D-F) TITAN results for individual species (or groups), presenting significant change points and 90% confidence limits; points are scaled in proportion to the magnitude of the response. Species codes on vertical axes: species number_ functional group_ rarity group, see text for codes.

All-night bat activity was detected in all three of our datasets (Fig 2A–2C). Moreover, there was a generally detectable bimodal activity pattern that roughly comprised the first four and the last hour of the night, which was evident to varying degrees across datasets. Although the overall capture frequency data supports this perception, the pattern is not consistent across rarity or functional categories, which were approximately evenly distributed along the night (S1 Fig). These findings were corroborated by the TITAN analysis for all datasets, in which most species did not clearly show any significant detection threshold (Fig 3A–3C). Species-rich Amazonian dataset presented notable exceptions, with activities of a common nectarivore and a group of uncommon omnivores associated with the beginning of the night (negative threshold), while several common and uncommon frugivores were positively associated with the end of the night (Fig 3D). Fewer species showed similar patterns in the *Atlantic forest* dataset, namely a common frugivore and a common sanguivore, with significant negative thresholds at the beginning of the night and a common nectarivore with a positive threshold at the end of the night (Fig 3E). In the *Cerrado* biome only a common nectarivore and a common frugivore were significantly associated with the beginning of the night, and there were no positive thresholds (Fig 3F). Community-thresholds found in all datasets (S2 Fig) should be interpreted cautiously, as they represent only 32%, 14% and 18% of all species tested for which a significant threshold was observed. All other species had no significant activity thresholds over the night, and these patterns were inconsistent with in terms of both rarity and functional category (Fig 3A, 3B and 3C, and S2 Table).

### Patterns of species richness and abundance

When comparing patterns of abundance, it becomes evident that larger sample sizes (12-hour strategy) result in more individuals captured, as predicted by the SA effect (S3 Fig). In the *Amazonia* dataset, 41% more individuals were sampled using this approach compared to the second most effective strategy. The comparable figures for the *Atlantic Forest* and the *Cerrado* were 33% and 27%, respectively. Comparing the two six-hour strategies, only the *Amazonia dataset* shows a substantial difference in captures, with the six-hour-B strategy yielding 10% more individuals. The *Atlantic Forest* sites showed only a 2% difference in efficiency in favor of the six-hour-B strategy, whereas the six-hour strategy yielded 4% more captures in the *Cerrado*.

**Fig 4:**
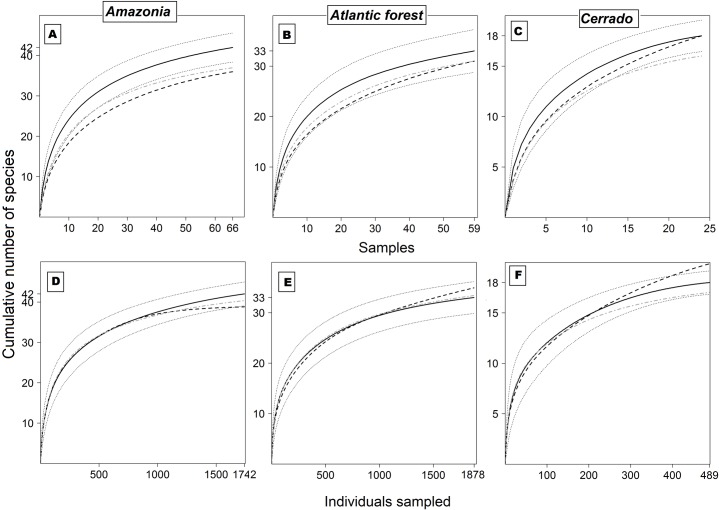
Species richness patterns. Sample-based species accumulation curves (A, B and C) and individual-based species accumulation curves (D, E and F) for the *Amazonia*, *Atlantic Forest* and *Cerrado* datasets. Solid black line: 12-hour sampling strategy (and 95% C.I.); dashed black line: six-hour sampling strategy; dot-dashed gray line: six-hour-B sampling strategy.

When examining species richness patterns, the predictions from the SA effect are generally held, with each dataset showing its own idiosyncrasies. Given an equivalent amount of sampling effort, sample-based accumulation curves showed more species recorded based on the 12-hour strategy than either one of the six-hour strategies for the *Amazonia* dataset (Fig 4A). Both the *Atlantic Forest* and *Cerrado* datasets showed similar patterns (Fig 4B and 4C). Nonetheless, extrapolated individual-based accumulation curves showed no significant differences between sampling strategies in terms of the number of species recorded for all datasets (Fig 4D, 4E and 4F). In terms of species richness, differences were largely related to sampling effort and the total number of individuals sampled, but once we extrapolate to a common number of individuals these differences are cancelled out. The exceptions were the *Amazonia* dataset, in which the six-hour strategy was significantly less species-rich after extrapolation, and the *Cerrado* dataset, in which the six-hour strategy was significantly more efficient than the 12-hour strategy based on extrapolations. In fact, considering the two half-night sampling strategies, the six-hour and six-hour-B strategies were more effective for the *Cerrado* and *Amazonia*, respectively, whereas there was no significant difference for the *Atlantic Forest* dataset (Fig 4D–4F).

### Patterns of species composition

**Fig 5:**
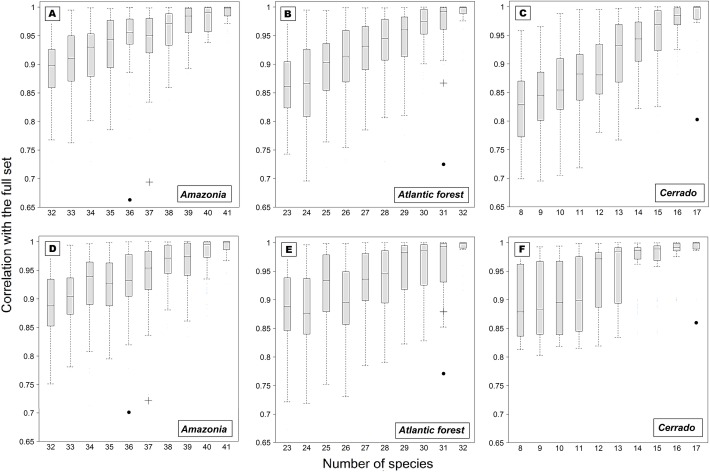
Species compositional patterns. Congruence between the six-hour sampling strategies and randomly generated subsamples (null model) of the entire community for each dataset. (A, B, C) Calculations were performed on the basis of the Jaccard dissimilarity using presence/absence matrices. (D, E, F) Calculations were performed on the basis of the Bray-Curtis dissimilarity using abundance matrices. Solid circle: six-hour sampling strategy; Black cross: six-hour-B sampling strategy.

Results on multivariate patterns of species composition indicate a clear effect of sampling strategies. Correlation values ranged between 0.66 and 0.87 for the Jaccard presence/absence matrices (*Amazonia*: “six-h” *r* = 0.663, *P* < 0.01; “six-h-B” *r* = 0.694, *P* < 0.01; *Atlantic Forest*: “six-h” *r* = 0.725, *P* < 0.01; “six-h-B” *r* = 0.867, *P* < 0.01; *Cerrado*: “six-h” *r* = 0.804, *P* < 0.01), and between 0.70 and 0.88 for the abundance-based Bray-Curtis matrices (*Amazonia*: “six-h” *r* = 0.701, *P* < 0.01; “six-h-B” *r* = 0.722, *P* < 0.01; *Atlantic Forest*: “six-h” *r* = 0.771, *P* < 0.01; “six-h-B” *r* = 0.879, *P* < 0.01; *Cerrado*: “six-h” *r* = 0.860, *P* < 0.01). While all calculated *r-*statistics indicated a significant correlation, all sampling strategies had lower correlations with their respective entire community than the randomly generated datasets with an equivalent number of species (Fig 5A–5F). The only exception was the *Atlantic Forest* dataset, for which the six-hour-B strategy performed better with the Bray-Curtis abundance matrix than random datasets (Fig 5E). Also, the six-hour-B strategy *r-*statistics was not calculated for *Cerrado* dataset, since its original matrix subsample had too many zeros, which can be interpreted as very poor performance. For the two other datasets, the six-hour-B strategy always outperformed the initial six-hour strategy.

## Discussion

We have shown that, regardless of the study region, the common strategy of restricting sampling of Neotropical bat assemblages to only the first six hours of the night can only provide an incomplete sample of the entire assemblage. The magnitude of this representation deficit in these subsamples depended on which sampling parameters were considered and, above all, varied between study regions. The species-richer *Amazonia* and *Atlantic Forest* datasets were more severely affected than the *Cerrado* dataset, suggesting different roles of the hypothesized effects on the observed patterns. Even our alternative six-hour-B strategy (at the beginning and the end of a sample night) was unable to capture the whole picture for the study bat assemblages, although it performed better in the *Amazonia* and the *Atlantic Forest*.

All-night bat activity was detected in all three datasets and overall frequencies suggested bimodal activity patterns. Conversely, specific patterns in activity related to rarity and functional traits were very weakly supported by threshold analysis (Fig 3). As observed in other studies [11, 14, 20], some interspecific variation was detected in the timing of activity peaks over the nocturnal cycle, especially for *Amazonia* and the *Atlantic forest*, but these represented the minority of species (Fig 3A, 3B and 3C). Rather, most species had no significant changes in detection, resulting in the all-night patterns detected for whole assemblages. In other words, the proposed biological effect (DSAP) was indeed detected, but played a minor and variable role in shaping detectability patterns observed across regions.

From a quantitative standpoint, patterns of species richness and abundance corroborated more strongly the predictions of the sample area effect (Fig 4 and S3 Fig). As shown for other ecological phenomena, such as the species-area relationship, this effect is derived primarily from passive sampling of the local species pool, in that larger samples effectively receive more individuals than smaller samples, and ultimately contain more species [25, 49]. However, extrapolating smaller samples to a common number of individuals results in statistically similar richness results (Fig 4D, 4E and 4F).

Corroboration of this null hypothesis should not rule out the possibility of alternative hypotheses by simple post-comparison, when this could be experimentally tested [25]. This was the case of our qualitative analysis approach, whereby species composition varied between six-hour sampling strategies in relation to their respective dataset over full 12-hour samples. The representation performance of our sampling strategies was significantly lower than those predicted by our null species composition models given an equivalent number of species (Fig 5). This means that, whatever the six-hour sampling strategy used, the identity of the species that are effectively sampled will be inherently impacted by choices of sampling schedule. Furthermore, while we demonstrated the major influence of null sample-area effects, combination of these results also show that the less evident DSAP effects do impact species composition representativeness during 6-hour sampling schemes, resulting in observed differences between regions. For *Amazonia*, where both 6-hours strategies were under-representative (especially the first 6 hours), DSAP effects were slightly stronger, while the opposite holds in the *Cerrado*, where DSAP effects had a minimal impact on the first 6-hour strategy. For the *Atlantic Forest*, DSAP effects were of intermediate importance, and significantly minimized when adopting the alternative 6-hours-B strategy (as in the *Amazonia*). Although the effects of subsampling assemblages have been detected previously [14], our study is the first to effectively decompose their effects into the temporal variation in capture efficiency both qualitatively and quantitatively. We further show that these effects cannot be accurately predicted based on species life history traits, but rather emerge as assemblage-wide patterns, manly influenced by sample-area effects.

While the evidence we gathered does achieve the goal of identifying important processes acting on the system of interest, another important step would be to further identify other dominant processes [49]. Even when results corroborated both hypothesized effects, species differences in foraging periodicity can exert a marked effect on the composition of species sampled depending on the region and the sampling strategy selected. These results bring insights into the question of why bat activity patterns are so variable (e.g. [20, 50, 51, 52]). There are also strong implications to hypothesis testing on, for example, the effects of anthropogenic habitat disturbance on vertebrate communities, particularly when habitat structure variably affects the circadian rhythms of different species (for a review: [53]).

Conversely, community composition correlation values between half-night and full-night sampling schedules were relatively high (66% to 87%) compared to other studies using similar methodological approaches [23, 54, 55]. In our view, the sufficiency in sampling representativeness in relation to the background community template must be carefully weighed against the principal study objectives and the trade-offs between further improving representation performance and logistical challenges that may arise from any given choice of sampling method.

### Enhancing sampling design

Marques and collaborators [13] proposed that moving nets each day would be a key strategy to minimize net shyness of bats, but only if that does not amount to losses in overall net time during a sampling expedition. If moving nets between sites results in loss of a sampling time (e.g. one day in a 24-day expedition), then translocating nets “costs” the same amount of sampling efficiency loss represented by net shyness, or approximately 42% of all individuals. Furthermore, they showed that this affects common species more severely than rare species, thereby concluding that if abundance is not the main focus of a study, daily net translocation could still be a reasonable sampling strategy even considering losses in sampling time.

Using similar quantitative evidence, we advocate that an investigator using a 12-hour sampling strategy would still lose a day of sampling time for obvious logistical reasons (even nocturnal fieldworkers must sleep!). However, by the end of a 24-day expedition, this campaign would yield 50%, 36% and 27% more individual captures if it were deployed in *Amazonia*, the *Atlantic Forest* or the *Cerrado*, respectively (S3 Fig). Furthermore, this would necessarily yield a significantly more species-rich sample in Amazonian expeditions (Fig 4). Likewise, if the 12-hour sampling approach is unfeasible for any reason, our novel six-hour–B strategy (at the onset and the end of the night) would still represent 10% higher efficiency and more species-rich samples, again if working in *Amazonia* (Fig 4). If, however, we place greater weight on qualitative species profiles, then sampling schedules covering the entire crepuscular, night, and pre-dawn 12-h period, or at least the beginning and the end of this period, becomes a much more logical strategy, since we maximize the probability of sampling different sets of species in all three forest and nonforest biomes investigated. These proposed strategies would allow sampling designs to account for the effects of both net avoidance and differential activity patterns, optimizing sampling efficiency considering logistical constraints, especially in species-rich bat assemblages such as those in *Amazonia*.

In summary, we presented detailed quantitative and qualitative information on the effects of selecting different sample size strategies when studying neotropical bats. Characterization of study assemblages were somehow affected by marked variation in detectable activity patterns across species in all three biomes examined here. On the other hand, the overall influence of temporal net deployment was variable between regions, and often fairly modest (r = 0.88 and 0.86 for the *Atlantic Forest* and *Cerrado*, respectively). Bat biologists planning study designs can therefore better decide on the most appropriate context-dependent sampling strategy, thereby optimizing resources for their study region.

## Supporting information

S1 Fig **Night-time bat activity patterns for the (A, B) *Amazonia*, (C, D) *Atlantic forest*, and (E, F) *Cerrado* datasets.** Stacked bar graphs showing frequency of captures during each hour throughout the entire night-time gradient, with species categorized into Rarity (A, C, E) and Functional groups (B, D, F).
(TIF)

S2 Fig TITAN´s Community Thresholds. Community-wide positive and negative thresholds, depicting cumulative sums of z-scores obtained in TITAN. The Environmental Gradient on X-axis refers to the 12 hours in the Nocturnal Cycle (1800h to 0600h).
(TIF)

S3 Fig Species abundance patterns. Cumulative number of individuals captured disaggregated by different sampling strategies for each of the three datasets. Solid black line: 12-hour sampling strategy; dashed black line: six-hour sampling strategy; Gray dot-dashed line: six-hour-B sampling strategy.
(TIF)

S1 Table Proportion of the variation in the data explained by the Principal Coordinates Analysis (PCoA). (XLSX)

S2 Table List of species with significant thresholds in activity detected using TITAN. Taxonomic, functional and rarity traits, detected thresholds (hour of the sampling night), associated 90% confidence limits, response (negative response: detection decreases after threshold; positive response: detection increases), IndVal z-score, median of z-score magnitude across all bootstrap replicates, IndVal statistic (scaled 0–100%), purity and reliability scores (see material and methods).
(XLSX)

S3 Table Primary data. (XLSX)
