# Supplementary figures and images for: Enhancing sampling design in mist-net bat surveys by accounting for sample size optimization
Source: PLoS One. 2017 Mar 23;12(3):e0174067. doi: 10.1371/journal.pone.0174067 (PMC5363843; doi:10.1371/journal.pone.0174067)

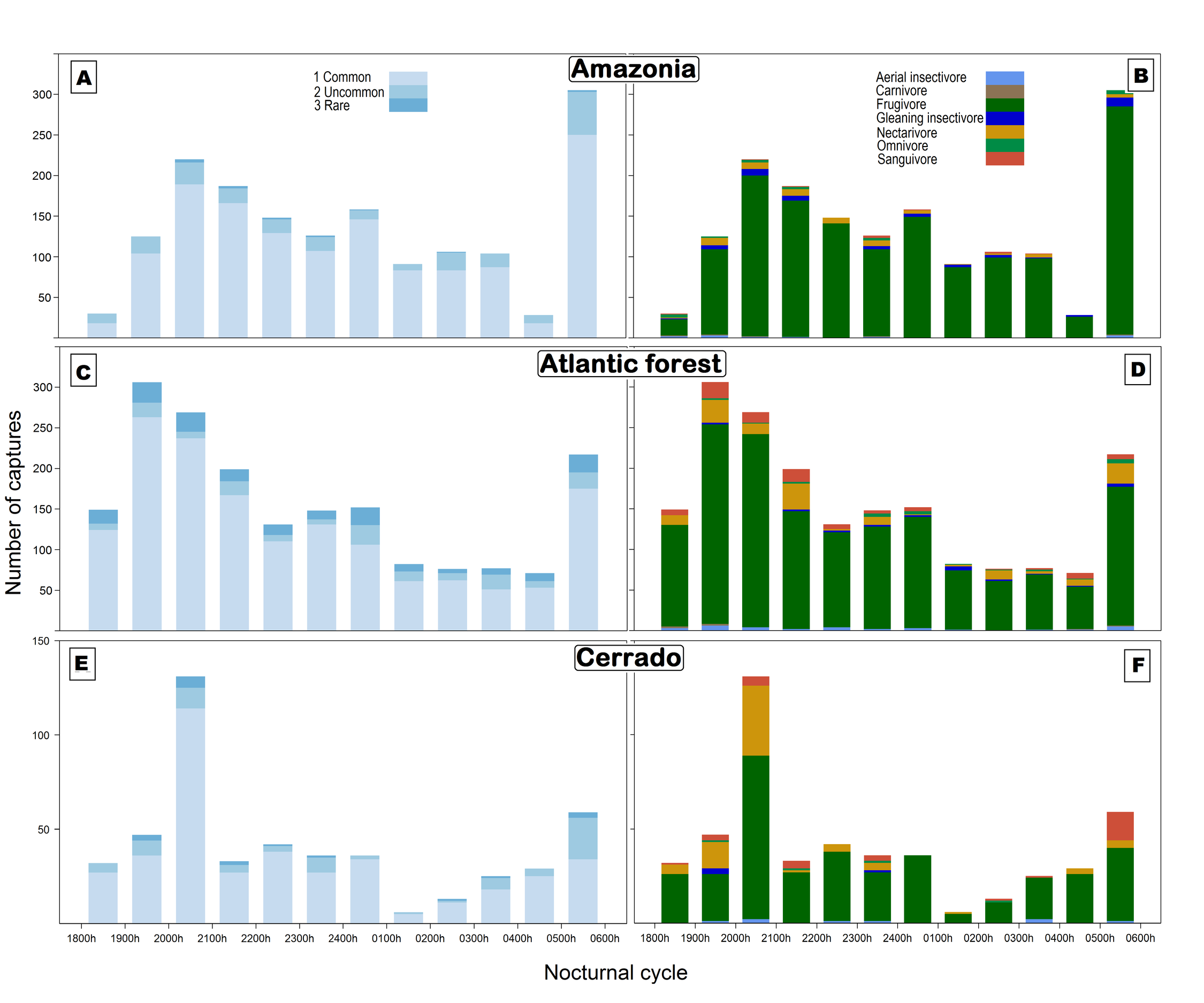

Supplement: S1 Fig — Night-time bat activity patterns for the (A, B) Amazonia, (C, D) Atlantic forest, and (E, F) Cerrado datasets. Stacked bar graphs showing frequency of captures during each hour throughout the entire night-time gradient, with species categorized into Rarity (A, C, E) and Functional groups (B, D, F). (TIF) [file pone.0174067.s001.tif]

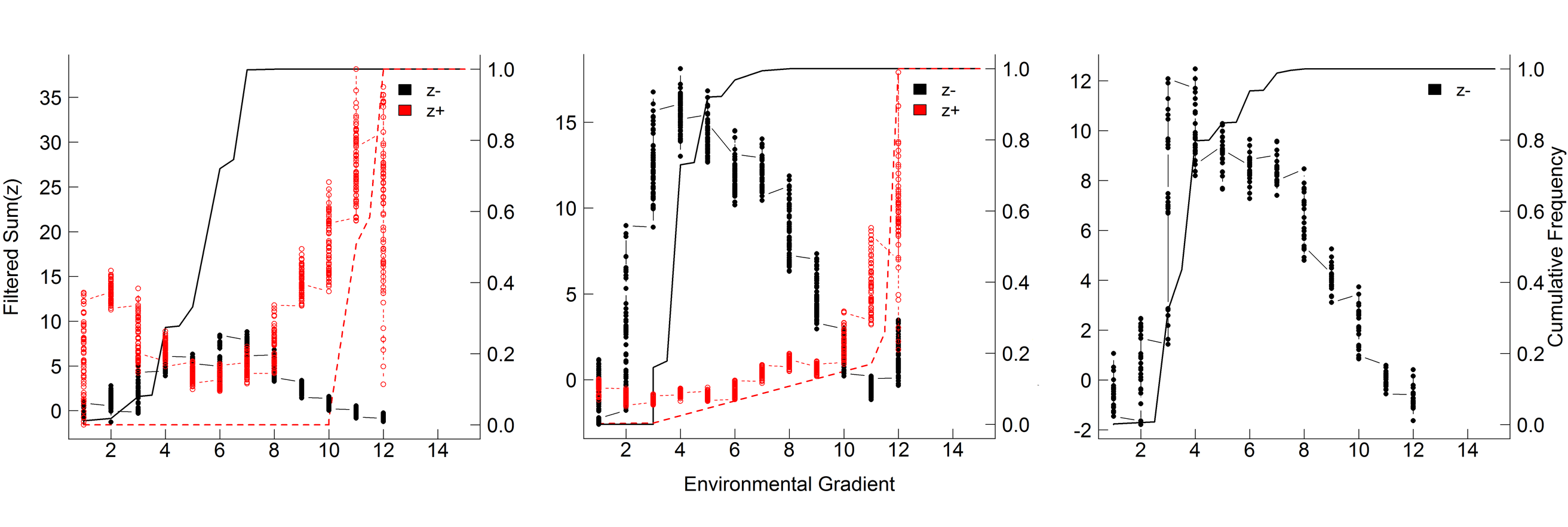

Supplement: S2 Fig — Community-wide positive and negative thresholds, depicting cumulative sums of z-scores obtained in TITAN. The Environmental Gradient on X-axis refers to the 12 hours in the Nocturnal Cycle (1800h to 0600h). (TIF) [file pone.0174067.s002.tif]

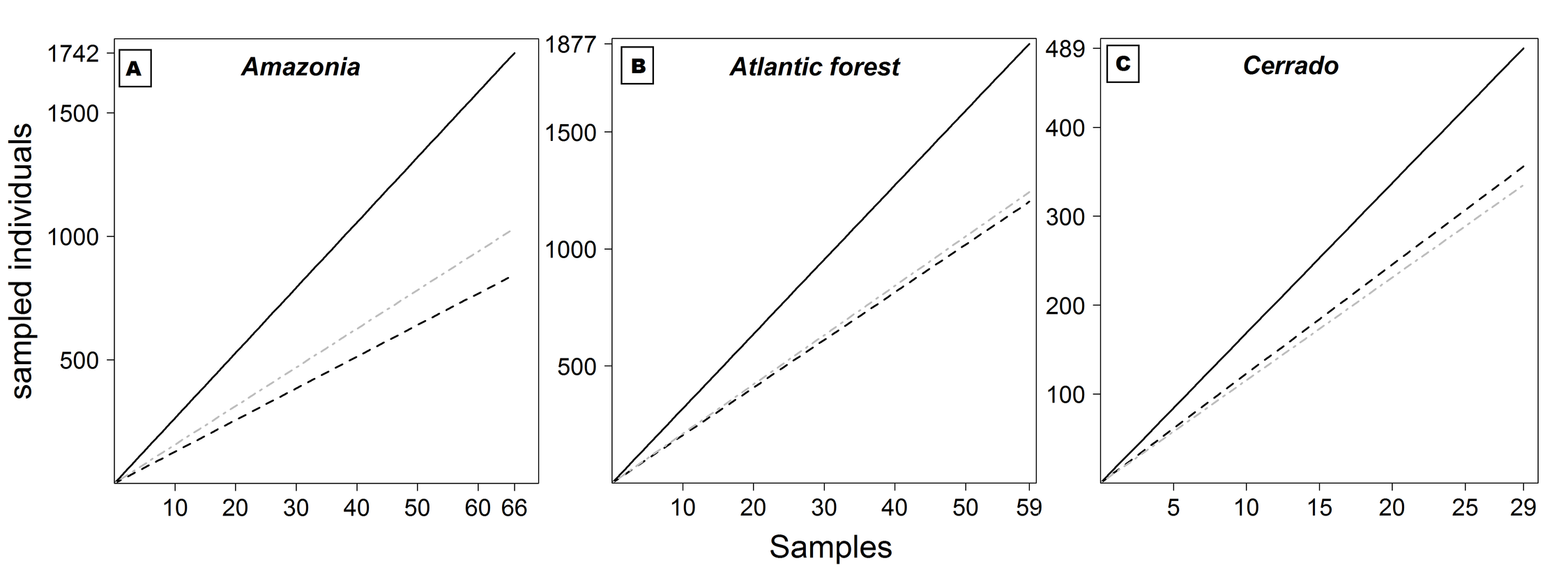

Supplement: S3 Fig — Cumulative number of individuals captured disaggregated by different sampling strategies for each of the three datasets. Solid black line: 12-hour sampling strategy; dashed black line: six-hour sampling strategy; Gray dot-dashed line: six-hour-B sampling strategy. (TIF) [file pone.0174067.s003.tif]
